# Tomato Fruit Detection Using Modified Yolov5m Model with Convolutional Neural Networks

**DOI:** 10.3390/plants12173067

**Published:** 2023-08-26

**Authors:** Fa-Ta Tsai, Van-Tung Nguyen, The-Phong Duong, Quoc-Hung Phan, Chi-Hsiang Lien

**Affiliations:** 1 Department of Mechanical Engineering, National United University, Miaoli 36002 Taiwan; fatatsai@nuu.edu.tw (F.-T.T.); nguyenvantung7879@gmail.com (V.-T.N.); 2 Department of Mechanical Engineering, HCMC University of Technology and Education, Ho Chi Minh City 700000, Vietnam

**Keywords:** tomato fruit detection, convolutional neural network, Yolov5

## Abstract

The farming industry is facing the major challenge of intensive and inefficient harvesting labors. Thus, an efficient and automated fruit harvesting system is required. In this study, three object classification models based on Yolov5m integrated with BoTNet, ShuffleNet, and GhostNet convolutional neural networks (CNNs), respectively, are proposed for the automatic detection of tomato fruit. The various models were trained using 1508 normalized images containing three classes of cherry tomatoes, namely ripe, immature, and damaged. The detection accuracy for the three classes was found to be 94%, 95%, and 96%, respectively, for the modified Yolov5m + BoTNet model. The model thus appeared to provide a promising basis for the further development of automated harvesting systems for tomato fruit.

## 1. Introduction

Tomatoes not only have succulent flesh and extreme sweetness, but they also contain many vitamins (C, E, and B), good acids (organic acids, phenolics, and chlorophyll), and many essential minerals that keep the human body in good health [1,2]. Besides the role tomatoes play in meeting daily nutrition needs, they also have a high economic value and are an important contributor to many local and national economies [3]. Nonetheless, the tomato farming industry faces a major challenge in that manual harvesting is labor intensive and inefficient. Consequently, the harvesting process is expensive, which increases the final cost to the consumer, and slow, which delays the delivery of the product to the market and reduces its aesthetic and nutritional value.

Many methods have been proposed for automating the tomato harvesting process [4,5,6,7]. Zhang et al. [8] developed a deep-learning model for tomato classification with an accuracy of 91.9% and a recognition time of just 0.01 s per 100 images. Taqi et al. [9] developed a robot system for cherry tomato harvesting in both private households and agricultural greenhouses. Zu et al. [10] used a Mask R-CNN [11] network model to detect and segment immature tomatoes on the vine based on the color of the tomato leaves. It was shown that the model achieved an F1 score of 92% and thus provided a reliable approach for automating the harvesting process. In general, the methods proposed in [4,5,6,7,8,9,10,11] have the potential to improve the tomato yield and go some way to alleviating the labor shortage problem caused by the COVID-19 pandemic [12,13]. However, these existing automated fruit harvesting methods still encounter challenges related to harsh environments and environmental interference, leading to compromised detection accuracy.

In recent years, artificial intelligence (AI) has been widely applied in many fields, including automobiles [14], the economy [15], and agriculture [16,17]. AI provides many important benefits, such as digital assistance, objective decision-making, improved speed and efficiency, reduced potential for human error, and 24/7 availability. As computer technology continues to advance, the detection and classification performance of AI models have improved accordingly, with the typical error rate in image recognition reducing from 29% in 2010 to less than 3% in 2017 [18]. Many AI methods have been developed for rapid object detection in real-time with high accuracy and minimal error. Some of the most well-known models include Region-based Convolutional Neural Network (R-CNN) [19], Faster R-CNN [20], Region-based (R-FCN) [21], Single-Shot Multi-Box Detector (SSD) [22], and You Only Look Once (YOLO) [23]. Compared to other methods, Yolo has a particularly fast recognition speed and high accuracy due to its end-to-end training. Hence, it is one of the most commonly used methods and has been successfully employed in many applications, including tomato detection [24,25].

Many different versions of the Yolo model have been developed over the years, including Yolov1, Yolov2, and Yolov3 [26]. The goal of Yolov1 (i.e., the original Yolo model) is to both detect and classify target objects in the image. This is achieved by treating the object detection problem as a regression problem. However, while Yolov1 performs well for large objects, it has problems recognizing small objects. Yolov2 (or Yolo9000) not only overcomes this problem by using the new training method and the anchor box accordingly, but it also has a speed similar to that of Faster R-CNN. Yolov3 has the same architecture as Yolov2 but replaces the softmax operation with logistic classifiers or uses the Darknet-53 structure as the backbone [27]. Bochkovskiy et al. [28] proposed a Yolov4 model, which combines the quintessence of research in object recognition detection in order to improve the speed and accuracy of the recognition process. Jocher et al. [29] presented a Yolov5 model with both a faster computational speed and a more straightforward implementation. Many versions of the Yolov5 model have been subsequently developed, including Yolov5n, Yolov5s, Yolov5m, Yolov5l, and Yolov5x, where the models differ mainly in their configurations and accuracy.

The literature contains many Yolo-based models for the detection of fruit products, such as oil palm fruit [30] and mango [31]. Liu et al. [32] presented an enhanced Yolov3 model for the identification of tomatoes in complex environments characterized by illuminance variations and object occlusion. The proposed model was shown to have an accuracy of up to 94.48% under ideal conditions. Guan et al. [33] designed a robot system based on Yolov5 for picking tomatoes on the vine. It was shown that the average recognition time of a single frame image was just 104 ms, which rendered the system suitable for real-time automatic picking applications. Egi et al. [34] presented a method for counting three classes of tomato fruit (ripe, green, and flowers) using Yolov5 and Deep Sort deep-learning algorithms. The method achieved detection accuracies of 85%, 99%, and 50% for the three classes, respectively.

In general, the results presented in [34] confirm the feasibility of improving the detection performance of the Yolo model through its integration with a further CNN. However, the existing Yolo-based methods may still face limitations in terms of accuracy and robustness, particularly in challenging conditions. The reliance on standard Yolo models may not fully address the nuanced complexities posed by varying lighting, occlusion, and fruit characteristics, which can lead to suboptimal performance. These drawbacks underscore the need for further improvements and modifications to enhance the automated tomato fruit detection process. Accordingly, the present study integrated the Yolov5m model with three other CNNs, namely BoTNet, ShuffleNet v2, and GhostNet. The classification performance of the three models was then compared with that of the original Yolov5m model for three classes of tomato fruit: ripe, immature, and damaged. Overall, the results showed that, among the various models, the modified-Yolov5m-BoTNet model provided the best detection performance with a mean average precision (mAP) of 94 over the three classes of tomatoes.

## 2. Results and Discussion

**Figure 1:**
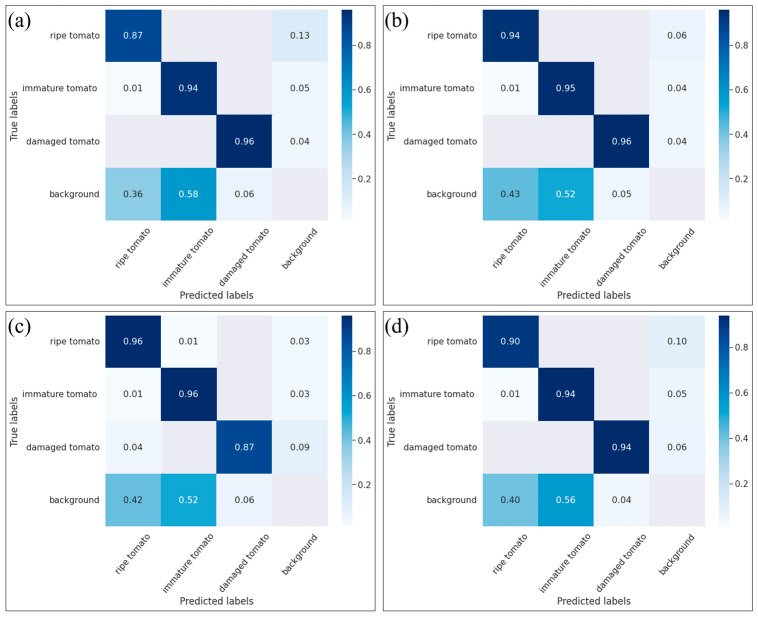
Confusion matrices for the (**a**) Yolov5m model, (**b**) modified-Yolov5m-BoTNet model, (**c**) modified-Yolov5m-ShuffleNet v2 model, and (**d**) modified-Yolov5m-GhostNet model.

Figure 1 shows the confusion matrices for the detection results of the four models. As shown in Figure 1a, the Yolo5m model had high detection accuracies of 94% and 96% for immature and damaged tomatoes, respectively. However, the detection accuracy for ripe tomatoes was only 87%. For the modified-Yolov5m-BoTNet model, the detection accuracy for ripe tomatoes increased to 94% (Figure 1b), representing an improvement of 7% over the original Yolo5m model. Moreover, the detection accuracies for immature and damaged tomatoes were 95% and 96%, respectively. Thus, the modified-Yolov5m-BoTNet model provided good detection accuracy for all three classes of tomatoes. The modified-Yolov5m-ShuffleNet model had a high detection accuracy of 96% for both ripe and immature tomatoes (Figure 1c). However, the detection accuracy for damaged tomatoes was just 87%. The poor detection performance can be attributed to the compact backbone structure of the ShuffleNet v2 network, which contains only 2.2 M parameters. Finally, the modilfied-Yolov5m-GhostNet model had detection accuracies of 90%, 94%, and 94% for ripe, immature, and damaged tomatoes, respectively (Figure 1d). Thus, the detection accuracy for ripe tomatoes increased by 3% compared to the original Yolov5m model. Overall, the results presented in Figure 1 confirm that the modified-Yolov5m-BoTNet model provided the best overall detection performance of the four models, closely followed by the modilfied-Yolov5m-GhostNet model.

**Figure 2:**
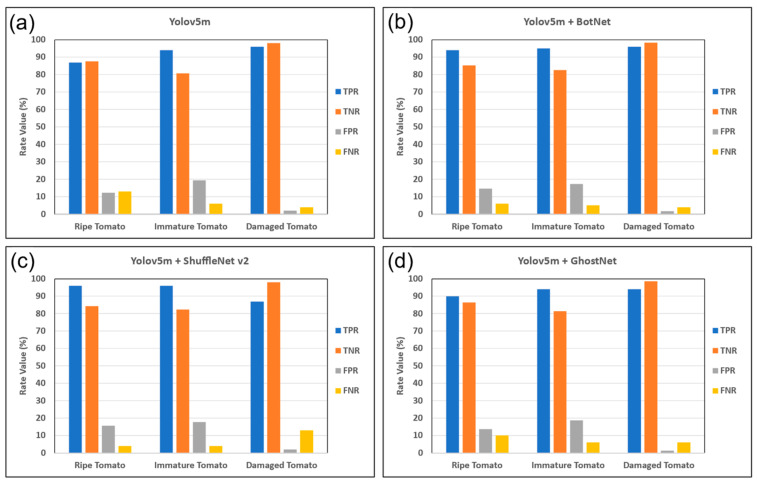
TPR, TNR, FPR, and FNR performance of (**a**) Yolov5m, (**b**) modified-Yolov5m-BotNet, (**c**) modified-Yolov5m-ShuffleNet v2, and (**d**) modified-Yolov5m-GhostNet.

Figure 2 shows the TPR, TNR, FPR, and FNR values of the four models. As shown, the modified-Yolov5m-BotNet model had the best TPR performance over the three classes of tomatoes, with a value in the range of 94–96%. All four models had a low FPR, with values in the range of 2–19%. The FNR rates of the modified-Yolov5m-BotNet model were the lowest among all the models, with values in the range of 4–6%. All four models had high TNR values ranging from 80 to 82% for both ripe and immature tomatoes. Moreover, all four models achieved a TNR value close to 98% for damaged tomatoes.

**Figure 3:**
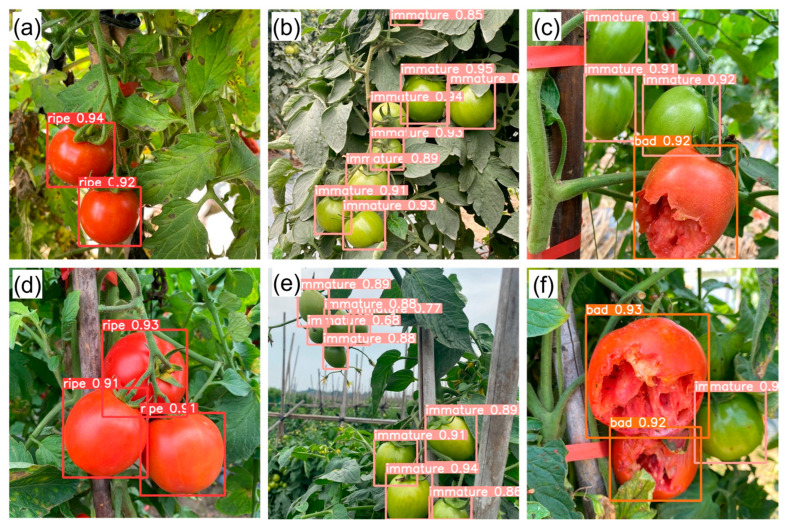
Real-world detection results obtained using the modified-Yolov5m-BoTNet model for: (**a**) ripe tomatoes, (**b**) immature tomatoes, (**c**) immature and damaged tomatoes, (**d**) ripe tomatoes, (**e**) immature tomatoes, and (**f**) damaged and immature tomatoes.

**Figure 4:**
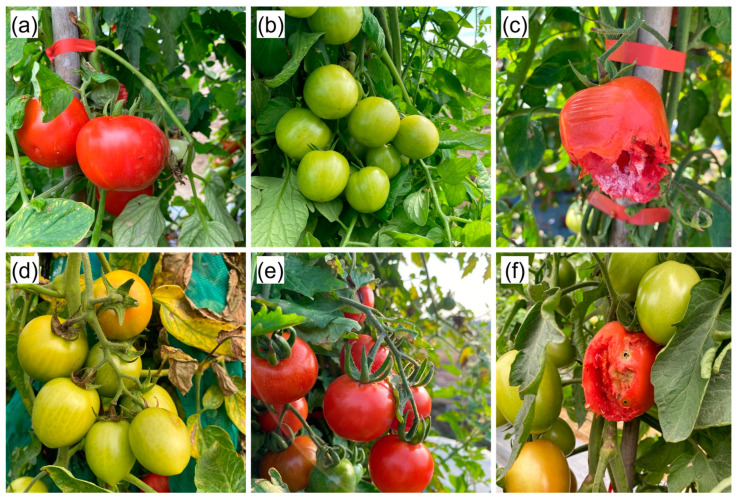
Typical normalized tomato images: (**a**) ripe tomatoes at 6:00 am, (**b**) immature tomatoes at 11:00 am, (**c**) damaged tomatoes at 12:00 pm, (**d**) immature tomatoes at 3:00 pm, (**e**) ripe tomatoes at 5:00 pm, and (**f**) immature and damaged tomatoes at 6:00 pm.

Figure 3 illustrates the detection results obtained using the modified-Yolov5m-BoTNet model in typical real-world scenarios (e.g., those shown in Figure 4). It is seen that the detection varied widely in the range of 70–90%, depending on the size of the target within the image, the color contrast of the target relative to the background, and the occlusion (or otherwise) of the fruit. When the fruit was located far from the camera (Figure 3e), the detection accuracy had a relatively low value of 70–80%. However, when the fruit was clear and non-occluded, the detection accuracy increased to as much as 95% (Figure 3a,b,d). Furthermore, even for occluded fruit, the detection accuracy had a high value of 90–93% (see Figure 3c,f).

## 3. Materials and Methods

### 3.1. Dataset for Training

A total of 1508 images of tomatoes were acquired from tomato farms in Miaoli County in western Taiwan and the Asian Vegetable Research and Development Center (AVRDC) in Tainan, south Taiwan. The images were obtained manually using a mobile phone (iPhone 11) and had a size of 3024 × 4032 pixels, a bit depth of 24, and a resolution of 72 dpi. To maintain consistency and reliability, a meticulous camera protocol was adhered. Images were acquired from a standardized distance of approximately 0.5 m, ensuring minimal distortion and maintaining consistent object proportions. The camera was positioned perpendicular to the plane of the fruit to mitigate any angular discrepancies. To increase the robustness of the training model, the images were acquired at different times of the day to achieve various illumination conditions and degrees of contrast between the targets (the tomatoes) and the background (the vine). The images were normalized to a size of 640 × 640 pixels to fit the Yolov5m model and improve the consistency of the training samples. It is noted that, in practical application, a commercial camera embedded in a vehicle might be used to capture the image instead of manually by an iPhone. Thus, the image distance and rotations might be changed. CNN’s-Yolo based models, such as many other object detection algorithms, are sensitive to variations in image distance and rotation. YOLO models are designed to detect objects within images by dividing the image into a grid and predicting bounding boxes and class probabilities for each grid cell. This design allows YOLO to identify objects in different positions within an image. To address these sensitivity issues, the training experiment with data augmentation techniques shown in Section 3.7 helped the model to become more invariant to these variations.

Figure 4 presents typical normalized images of the three tomato classes (ripe, immature, and damaged) at various times of the day from 6:00 am to 6:00 pm. The tomatoes in the 1508 images were labeled with the appropriate classes using LabelImg open-source software version 1.8. In total, the images contained 2868 ripe, 3712 immature, and 885 damaged tomatoes. The data (200 MB) are available upon request to interested researchers for further analysis and validation.

### 3.2. Yolov5 Model

**Figure 5:**
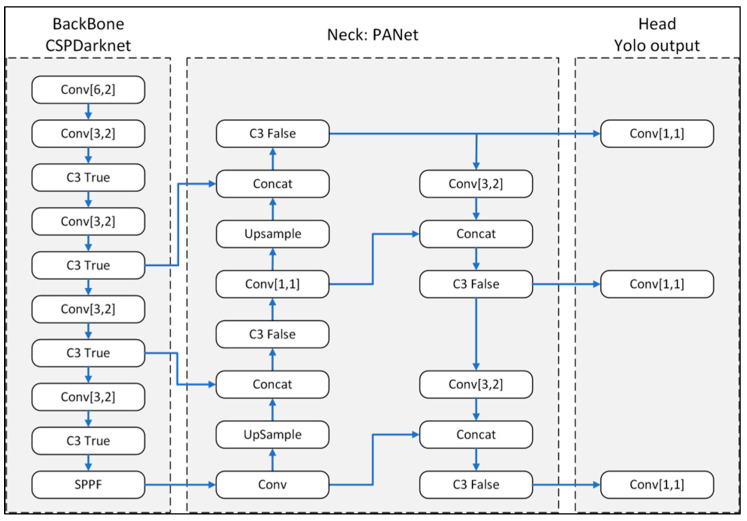
Basic structure of the Yolov5m model.

Figure 5 shows the basic structure of the Yolov5m model consisting of three blocks, namely the backbone, neck, and head. The data are first input to the backbone, which is implemented with CSPDarrknet. The backbone consists mainly of a C3 module (cross-stage partial network bottleneck with 3 convolutions), which improves on the CSPResBlock module in Yolov4 through the use of a single convolution layer and thus increases the training speed and reduces the number of training parameters. The backbone additionally incorporates a Spatial Pyramid Pooling Fast (SPPF) module. The SPPF module is similar to that of the Spatial Pyramid Pooling (SPP) module in Yolov4. However, SPPF is two times faster than SPP and employs kernels of different sizes in order to increase the receptive field and enable the processing of input images with a greater size variation. The neck block (PANet) uses multiple convolution and concatenation (Concat) blocks to extract feature pyramids from the input images. The neck also contains an Upsample module to enhance the subsample recognition accuracy. Finally, the head block, also known as the object detection module, predicts the coordinates of the bounding box (position, dimensions) for each target object of interest in the input image.

### 3.3. BoTNet Transform Model

**Figure 6:**
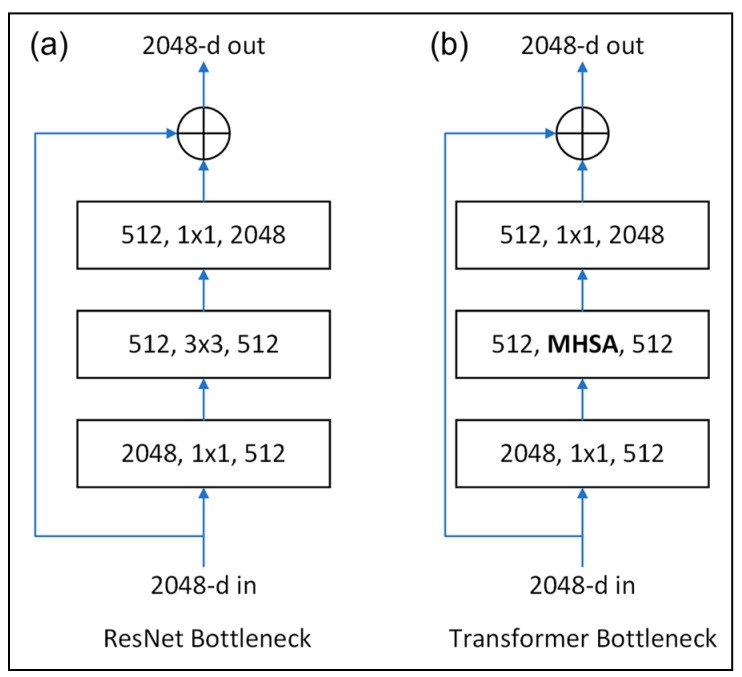
(**a**) ResNet bottleneck and (**b**) BoTNet transformer bottleneck.

Srinivas et al. [35] proposed a BoTNet transform model with the ability to detect and classify not only whole objects of different classes, but also segments of objects of the same class. The model was based on the bottleneck block structure used in ResNet [36]. As shown in Figure 6a, the ResNet bottleneck block comprises three consecutive convolution operations, including a 1 × 1 convolution to reduce the number of feature depths, a 3 × 3 convolution to extract features from images, and a 1 × 1 convolution to increase the number of feature depths relative to the original input. In the bottleneck architecture, the input values changed progressively from 2048 to 512 and then back to 2048. In other words, the output value was equal to the original number of dimensions. As shown in Figure 6b, the BoTNet model [35] was implemented using a transformer bottleneck block, in which the 3 × 3 convolution operation in ResNet50 was replaced with a Multi-Head Self-Attention (MHSA) module. It was shown in [35] that the combined use of convolutions and the MHSA improved the average precision (AP) of the mask by around 1.2% for the Common Objects in Context (COCO) instance segmentation benchmark.

**Figure 7:**
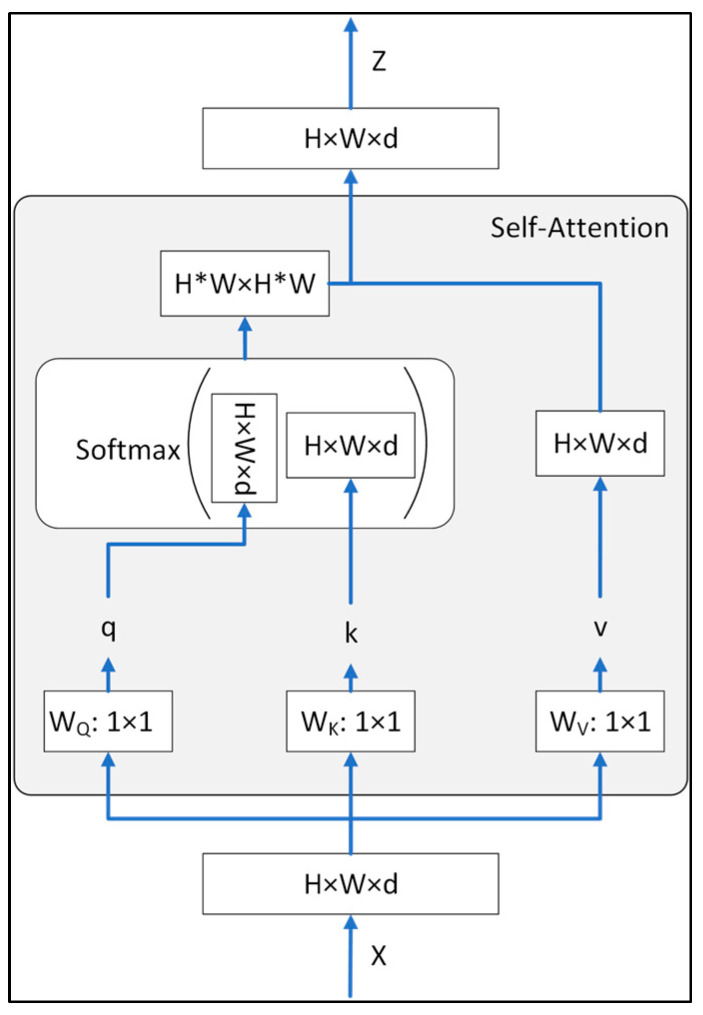
Multi-head self-attention module.

Figure 7 shows the structure of the MHSA module. As shown, the module extracts long-range structural data from the input images [37,38,39,40]. The MHSA is used to connect components in the highest feature map. As a result, it provides access to a receptive field that includes all of the input images, and hence, the precision of a particular pixel is influenced by every input pixel. The MHSA module has three inputs, named the matrices of the queries (Q), keys (K), and values (V), denoted as Wq, Wk, and Wv, respectively. The self-attention module is calculated separately in multiple heads before being combined through another embedding, and the independent self-attention outputs are then concatenated and linearly transformed into the expected dimension. The multiple self-attention headers allow self-attention to be paid to different parts of the sequence. The MHSA is calculated as:

(1) $$MutiHead(Q,K,V)=[head_{1},\ldots ,head_{h}{]W}_{0}$$

where $head_{i}=SelfAttention\left(QW_{i}^{Q},KW_{i}^{K},VW_{i}^{V}\right)$, and W are all learnable parameter matrices.

**Figure 8:**
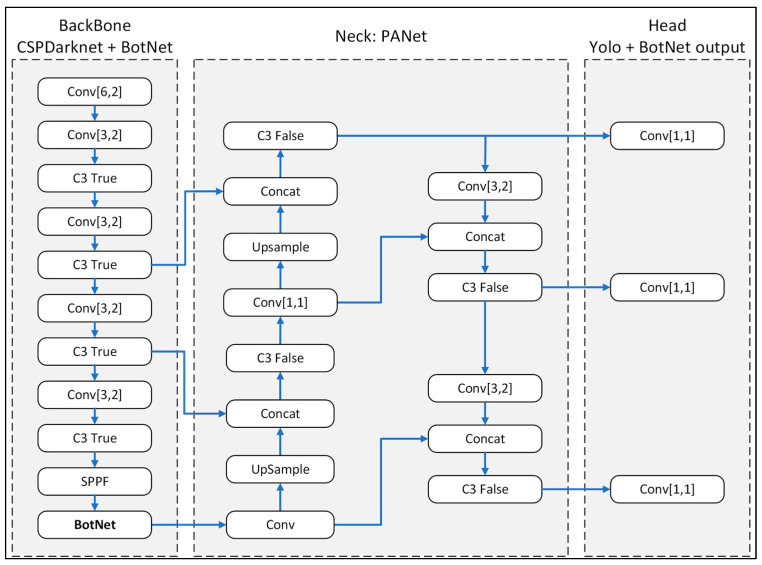
Modifed-Yolov5m-BotNet transform model.

In the present study, the BotNet transform model was integrated within the backbone of the original Yolov5m network, as shown in Figure 8. The BotNet module was added behind the SPPF block of the original structure to enhance the feature map recognition of the input image through the MHSA module. Thus, the accuracy of the object detection process was improved, while the training time was reduced.

### 3.4. ShuffleNet Model

**Figure 9:**
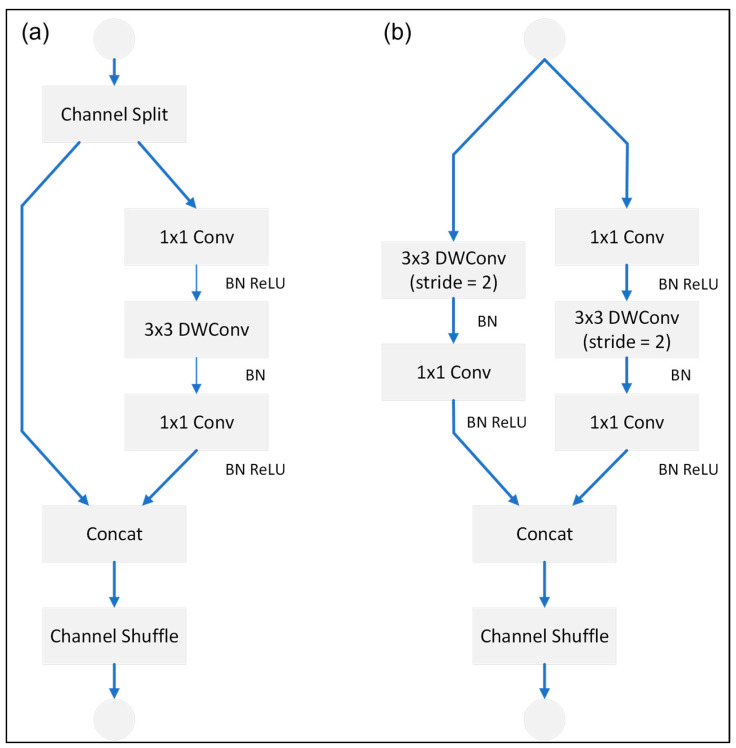
Shuffle units of ShuffleNet v2: (**a**) basic unit of ShuffleNet v2, and (**b**) shuffle unit used for spatial down-sampling.

Deep learning network models are becoming more and more accurate. However, this performance improvement is often obtained at the expense of a very large number of parameters, which increases the training cost and prevents their deployment on weak hardware devices, such as mobile devices. Several lightweight deep learning models have been developed to address this problem, including MobileNet, NASNETMobile, and ShuffleNet v2 [41,42,43]. ShuffleNet v2 is an extension of the original ShuffleNet model proposed in [44] with pointwise group convolutions, bottleneck structures, and channel shuffle operations, and it was designed to further optimize the efficiency of the original structure. ShuffleNet v2 contains multiple shuffle units, which repeat different strides for different numbers of times. Figure 9 illustrates the structure of the shuffle unit. As shown in Figure 9a, the shuffle unit network includes channel split, concatenation, and channel shuffle blocks. After the initial channel split operation, one of the branches passes directly to the concatenation block, while the other branch contains two 1 × 1 ungrouped convolutions and a depth-wise convolution (DWConv) block. The outputs of the two branches are merged with the Concat block to ensure the same number of inputs and outputs. The channel shuffle block then allows for information exchange between the two branches. Figure 9b shows the structure of the spatial down-sampling module in the shuffle unit network. As shown, the split channel block is omitted, thus minimizing the number of input parameters and improving the speed as a result.

**Figure 10:**
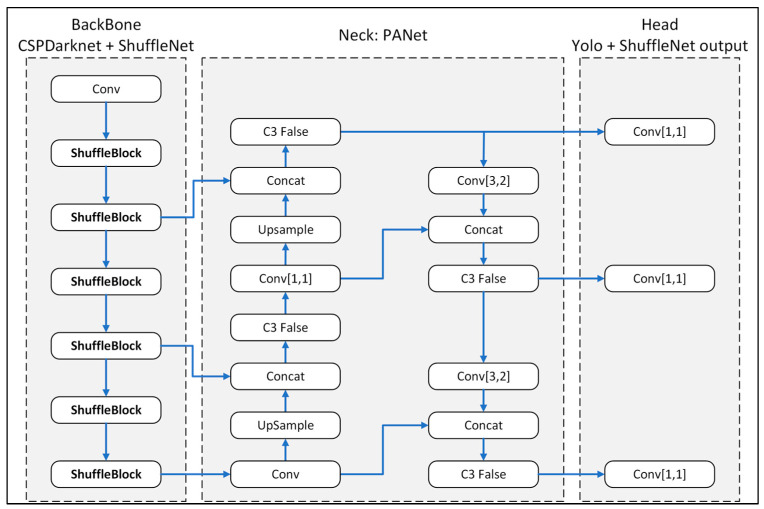
Modified-Yolov5m-ShuflfeNet model structure.

In the present study, ShuffleNet v2 was integrated with Yolov5m in order to improve the efficiency of the training process. In particular, the backbone structure of the original Yolov5m model was implemented using stacked Shuffle blocks in order to reduce the number of training parameters and giga floating point operations per second (GFLOPs) (see Figure 10).

### 3.5. GhostNet Model

**Figure 11:**
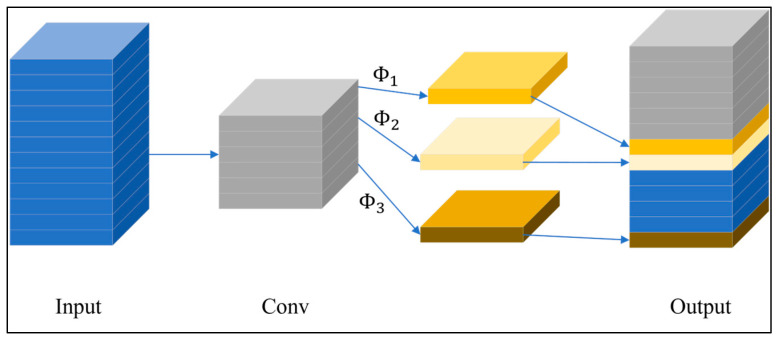
Structure of the Ghost module.

GhostNet [45] is a cheap CNN based on a stack of Ghost bottleneck layers. As shown in Figure 11, the first layer of the network comprises a convolutional layer with 16 filters. The next layer consists of multiple stacked Ghost bottleneck layers (Φ), while the last layer is a convolutional layer with a feature vector dimension of 1280. Notably, the GhostNet model uses linear transformation to maintain the feature map with the normal convolution output [46]. Furthermore, the model mainly uses low-level linear math to enhance the feature map and processing channel. Thus, although the network has many convolutional layers, it has a relatively low number of GFLOPs. Consequently, the GhostNet network provides a high detection accuracy, but it can still be implemented on weak hardware devices.

**Figure 12:**
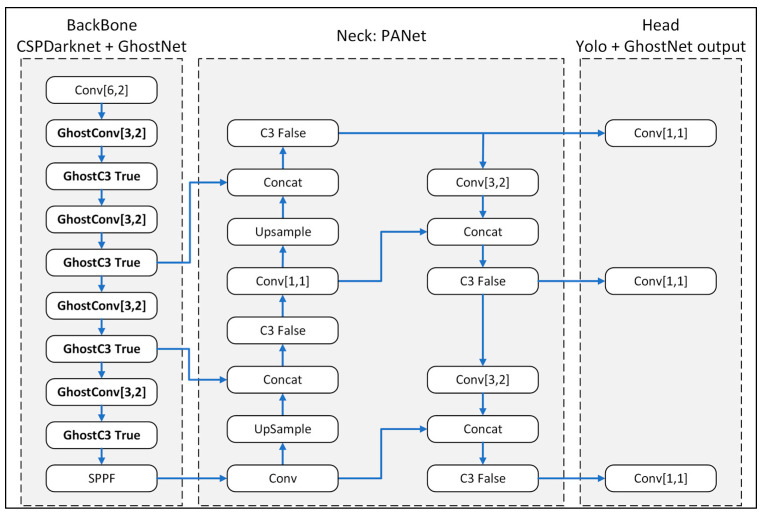
Modified-Yolov5m-GhostNet model backbone structure.

In this study, the GhostNet model was incorporated into the backbone of Yolov5m in order to increase the number of layers while simultaneously reducing the GFLOP score. As shown in Figure 12, the modified component of the backbone structure consisted of GhostC3 cross-stage partial network bottlenecks with three convolutions and GhostConv convolution blocks.

### 3.6. Evaluation Metrics

The performance of object classification models is generally evaluated using a confusion matrix, as shown in Table 1.

For object detection problems, such as that considered in the present study, the performance can be evaluated using the Intersection over Union (IoU) metric, defined as:

(2) $$IoU=\frac{Area\;of\;Overlap}{Area\;of\;Union}$$

where the area of overlap is the area of intersection between the predicted bounding box and the ground truth, while the area of union is the combined area of the predicted bounding box and the ground truth.

The performance of a classification model can be further evaluated using the precision and recall metrics, where the precision provides a measure of the prediction accuracy, and the recall gives a measure of the quantity of the finding ability. The precision and recall are defined, respectively, as:

(3) $$Precision=\frac{TP}{TP+FP}$$

(4) $$Recall=\frac{TP}{TP+FN}$$

The F1-score is the harmonic mean of the precision and recall and provides a more representative assessment of the precision than the precision or recall metric alone. The F1-score is defined as:

(5) $$F1=2\times \frac{Recall\times Precision}{Recall+Precision}$$

The average precision (AP) is the weighted sum of the precision at each threshold, where the weight is defined as the increase in recall between thresholds. In other words, the AP is calculated as,

(6) $$AP=\overset{k=n-1}{\underset{k=0}{\sum }}\left[Recall\left(k\right)-Recall\left(k+1\right)\right]*Precision\left(k\right)$$

where n is the number of thresholds.

The mAP (mean average precision) is the average AP value computed over the different classes, i.e.,

(7) $$mAP=\frac{1}{n}\overset{k=n}{\underset{k=1}{\sum }}AP_{k}$$

where AP_k_ is the AP of class k, and n is the number of classes.

Finally, from the confusion matrix, the following performance metrics can be derived:

(8) $$TPR=\frac{TP}{\sum Positive}=\frac{TP}{FN+TP}$$

(9) $$TNR=\frac{TP}{\sum Negative}=\frac{TP}{FN+TP}$$

(10) $$FPR=\frac{FP}{\sum Negative}=\frac{FP}{FP+TN}$$

(11) $$FNR=\frac{FN}{\sum Positive}=\frac{FN}{FN+TP}$$

where TPR is the true positive rate, TNR is the true negative rate, FPR is the false positive rate, and FNR is the false negative rate.

### 3.7. Training Data

**Figure 13:**
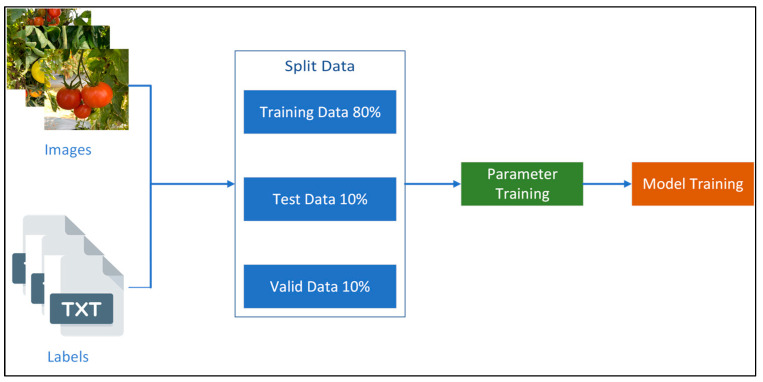
The chart shows the ratio data for training.

As shown in Figure 13, the 1508 annotated images were randomly separated into a training dataset (80%), a testing dataset (10%), and a validation dataset (10%). For each model, the training process was performed using the parameters shown in Table 2. The hardware implementation details of the training process are shown in Table 3.

**Figure 14:**
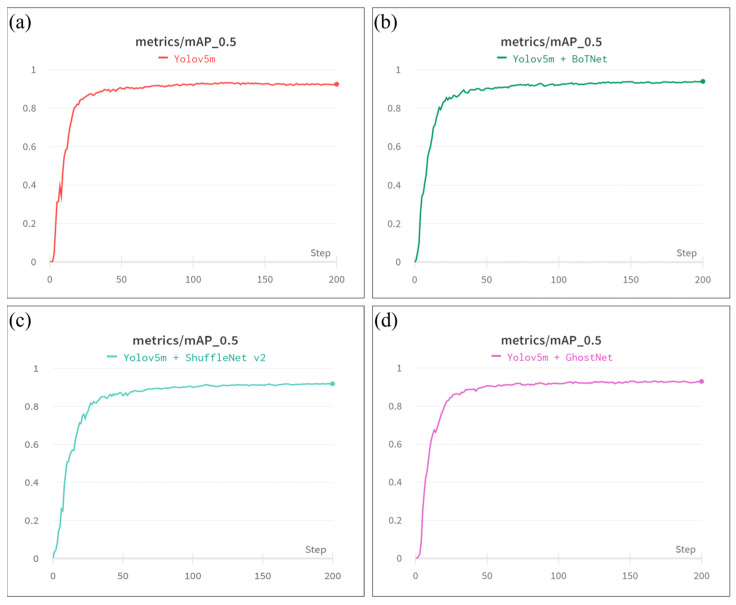
mAP values of: (**a**) Yolov5m, (**b**) modified-Yolov5m-BoTNet, (**c**) modified-Yolov5m-ShuffleNet, and (**d**) modified-Yolov5m-GhostNet.

Figure 14 shows the evolution of the mAP values of the original Yolov5m model and the three modified Yolov5m models over the training process. The training results for each model are summarized in Table 4. As shown, the original Yolov5m model achieved an mAP value of 0.92. The modified-Yolov5m-BoTNet model achieved an mAP value of 0.94, corresponding to a 2.17% improvement over the original model. For the modified-Yolov5m-ShuffleNet model, the mAP reduced to 0.92 (i.e., the same as the original model). Finally, for the modified-Yolov5m-GhostNet model, the mAP increased slightly to 0.93, an increase of 1.09% compared to the original Yolov5m model. Of all the models, the modified-Yolov5m-ShuffleNet v2 model had the smallest number of parameters (2.2 M) and the fastest training time (1.97 h). The ShuffleNet v2 model also had the smallest GFLOP score of 4.8, which is significantly smaller than that of the original Yolov5m model (47.9). The modified-Yolov5m-BoTNet and modified-Yolov5m-GhostNet models also had relatively lower GFLOP scores of 15.5 and 29.3, respectively. The F1 scores of the four models varied in from 0.84 to 0.87, indicating that all of the models achieved a high detection accuracy despite the complexity of the detection task, which was characterized by significant variances in color, size, and illumination.

## 4. Conclusions

Three CNN models based on modified Yolov5m networks with BoTNet, ShuffleNet v2, and GhostNet incorporated into the backbone structure, respectively, were proposed for the automatic detection of three classes of tomato fruit on the vine, namely ripe, immature, and damaged. The detection results showed that, among the various models, the modified-Yolov5m-BoTNet model achieved the highest mAP value of 0.94 across the three tomato classes. Moreover, the detection accuracy of the modified-Yolov5m-BoTNet model had a high value in the range of 94–96%. The modified-Yolov5m-GhostNet model also had a high accuracy of 90–94%. However, the original Yolov5m model and modified-Yolov5m-ShuffleNet v2 model had generally lower accuracies of 87–96%. The TNR and TPR values of all the models varied in the range of 82–98%, while the FPR and FNR values varied in the range of 2–19%. Compared with the original Yolov5m model, the proposed modified models (in particular, the modified-Yolov5m-BoTNet model and modified-Yolov5m-GhostNet model) had significantly improved detection accuracies for tomato fruit. Furthermore, the models have fewer parameters than the original model, and thus not only have a faster training time, but they can also be implemented on weak hardware devices such as smartphones and other mobile devices. Consequently, they have significant potential for the realization of reliable and low-complexity automated fruit harvesting systems. In the future, the development of a fully automated harvesting system is anticipated. A refining algorithm was built to accommodate diverse environmental conditions and fruit characteristics, ensuring seamless integration with real-world agricultural practices. Future endeavors will involve an in-depth exploration of the models’ adaptability to dynamic field conditions, encompassing factors such as lighting variations, weather influences, and diverse vine layouts.

## Figures and Tables

**Table 1:** Confusion matrix structure.

	Predicted Condition		
**True Condition**	P + N	PP	PN
	(Total Population)	(Predict Positive)	(Predict Negative)
	P	TP	FN
	(Positive)	(True Positive)	(False Negative)
	N	FP	TN
	(Negative)	(False Positive)	(True Negative)

Note that P denotes the actual total number of positive cases, and N is the total number of negative cases. Furthermore, true positive (TP) indicates that the model correctly predicts a positive case. Similarly, true negative (TN) indicates that the model correctly predicts a negative case. Meanwhile, false positive (FP) indicates that the model erroneously predicts a negative sample as positive, while false negative (FN) indicates that the model wrongly predicts a positive sample as negative.

**Table 2:** Training parameters.

Parameter	Value
Optimization	Adam
Batch size	32
Learning rate	0.0001
Decay	5 × 10^−5^
Drop out	0.1
Epochs	200
Image size	640 × 640 pixel
Augmentation hyperparameters	hyp.scratch-high.yaml

**Table 3:** Training system hardware.

CPU	GPU	Ram	Disk
2 × Xeon Processors @2.3 Ghz, 46 MB Cache	2 × Tesla T4 16 GB	16 GB	80 GB

**Table 4:** Summary of training results.

Model	Layer	Parameter	GFLOPS	mAP	F1 Score	Time (h)
Yolov5m	212	21.0 M	47.9	0.92	0.86	2.32
Modified-Yolov5m-BoTNet	162	6.7 M	15.5	0.94	0.87	1.98
Modified-Yolov5m-ShuffleNet v2	221	2.2 M	4.8	0.92	0.84	1.97
Modified-Yolov5m-GhostNet	378	14.2 M	29.3	0.93	0.87	2.44

## Data Availability

Data are available on request.
